# Defining Sarcopenia in a Population Based Study of Oldest Adults

**DOI:** 10.1093/geroni/igaf122.1218

**Published:** 2025-12-31

**Authors:** Josef Coresh, Shoshana Ballew, Jordan Weiss, Morgan Grams, B Gwen Windham, Lynne Wagenknecht, Marcus Goncalves, Hubert Leo

**Affiliations:** New York University, New York, New York, United States; New York University, New York, New York, United States; NYU Grossman School of Medicine, New York, New York, United States; New York University, New York, New York, United States; University of Mississippi Medical Center - The MIND Center, Jackson, Mississippi, United States; Wake Forest University School of Medicine, Winston-Salem, North Carolina, United States; New York University, New York, New York, United States; New York University, New York, New York, United States

## Abstract

Sarcopenia is common in older age and is associated with increased risk of mortality and other complications. Sarcopenia definitions vary, but consensus suggests the following should be present: (1) low muscle mass; (2) impaired strength; and (3) decreased performance. We sought to assess the agreement and correlates of candidate input variables in 6,515 Atherosclerosis Risk in Communities (ARIC) Study participants at visit 5 (2011-2013, age 66+ years). The input variables were fat free mass (FFM kg/m2) using bioimpedance analysis (BIA, lower limb leads), grip strength (grip, kg), and walking speed (WS, 4-meter walk, m/s). Age correlations were: -0.03 for FFM, -0.18 for grip and -0.23 for WS. Correlations between variables were 0.03 in men/-0.05 in women (FFM and grip), -0.05/-0.32 (FFM and WS) and 0.25/0.21 (grip and WS), respectively. Grip and WS were each strongly related to mortality and hospitalization. Having both weak grip and slow WS associated with a doubling in mortality risk (hazard ratio=2.3 (95% CI 2.0-2.6)) compared to having neither, supporting the sarcopenia definition put forward by the Sarcopenia Definitions and Outcomes Consortium. FFM association with mortality was weak and inconsistent across strata of grip and WS, suggesting limitations in the use of BIA in defining sarcopenia. In summary, we detail differences across the sarcopenia input variables by demographics, as well as their implications for risk associations. More robust measures of muscle mass (e.g. DXA vs. BIA with lower limb leads) may add value to sarcopenia definitions.

